# Extensive Bioinformatics Analyses Reveal a Phylogenetically Conserved Winged Helix (WH) Domain (Zτ) of Topoisomerase IIα, Elucidating Its Very High Affinity for Left-Handed Z-DNA and Suggesting Novel Putative Functions

**DOI:** 10.3390/ijms241310740

**Published:** 2023-06-27

**Authors:** Martin Bartas, Kristyna Slychko, Jiří Červeň, Petr Pečinka, Donna J. Arndt-Jovin, Thomas M. Jovin

**Affiliations:** 1 Department of Biology and Ecology, University of Ostrava, 710 00 Ostrava, Czech Republic; 2 Emeritus Laboratory of Cellular Dynamics, Max Planck Institute for Multidisciplinary Sciences, 37077 Göttingen, Germany

**Keywords:** Z-DNA, topoisomerase IIα, topoII, GTP, bioinformatics

## Abstract

The dynamic processes operating on genomic DNA, such as gene expression and cellular division, lead inexorably to topological challenges in the form of entanglements, catenanes, knots, “bubbles”, R-loops, and other outcomes of supercoiling and helical disruption. The resolution of toxic topological stress is the function attributed to DNA topoisomerases. A prominent example is the negative supercoiling (nsc) trailing processive enzymes such as DNA and RNA polymerases. The multiple equilibrium states that nscDNA can adopt by redistribution of helical twist and writhe include the left-handed double-helical conformation known as Z-DNA. Thirty years ago, one of our labs isolated a protein from *Drosophila* cells and embryos with a 100-fold greater affinity for Z-DNA than for B-DNA, and identified it as topoisomerase II (gene Top2, orthologous to the human UniProt proteins TOP2A and TOP2B). GTP increased the affinity and selectivity for Z-DNA even further and also led to inhibition of the isomerase enzymatic activity. An allosteric mechanism was proposed, in which topoII acts as a Z-DNA-binding protein (ZBP) to stabilize given states of topological (sub)domains and associated multiprotein complexes. We have now explored this possibility by comprehensive bioinformatic analyses of the available protein sequences of topoII representing organisms covering the whole tree of life. Multiple alignment of these sequences revealed an extremely high level of evolutionary conservation, including a winged-helix protein segment, here denoted as Zτ, constituting the putative structural homolog of Zα, the canonical Z-DNA/Z-RNA binding domain previously identified in the interferon-inducible RNA Adenosine-to-Inosine-editing deaminase, ADAR1p150. In contrast to Zα, which is separate from the protein segment responsible for catalysis, Zτ encompasses the active site tyrosine of topoII; a GTP-binding site and a GxxG sequence motif are in close proximity. Quantitative Zτ-Zα similarity comparisons and molecular docking with interaction scoring further supported the “B-Z-topoII hypothesis” and has led to an expanded mechanism for topoII function incorporating the recognition of Z-DNA segments (“Z-flipons”) as an inherent and essential element. We further propose that the two Zτ domains of the topoII homodimer exhibit a single-turnover “conformase” activity on given G(ate) B-DNA segments (“Z-flipins”), inducing their transition to the left-handed Z-conformation. Inasmuch as the topoII-Z-DNA complexes are isomerase inactive, we infer that they fulfill important structural roles in key processes such as mitosis. Topoisomerases are preeminent targets of anti-cancer drug discovery, and we anticipate that detailed elucidation of their structural–functional interactions with Z-DNA and GTP will facilitate the design of novel, more potent and selective anti-cancer chemotherapeutic agents.

## 1. Introduction

This report represents the convergence of two molecular biological “currents”. The first is the focus of this Special Issue of IJMS, the enzymatic activities discovered and denoted as “DNA topoisomerase(s)” by James Wang in 1979 [1] and extensively characterized structurally and functionally since then (reviewed in Refs. [2,3,4]). The second is the family of “non-B” DNA structures [5] deemed to intervene in cellular processes [6], one of which is the “Z-DNA” double helix with a left-handed helical sense, first proposed in 1970–1972 based on solution studies of alternating pur-pyr d[G-C] sequences [7,8] and confirmed by X-ray crystallography a decade later [9,10]. The structures and generation of Z-DNA and related Z-RNA have been reviewed recently [11,12,13], as have the biological roles attributed to them [14,15,16,17,18,19].

**Figure 1:**
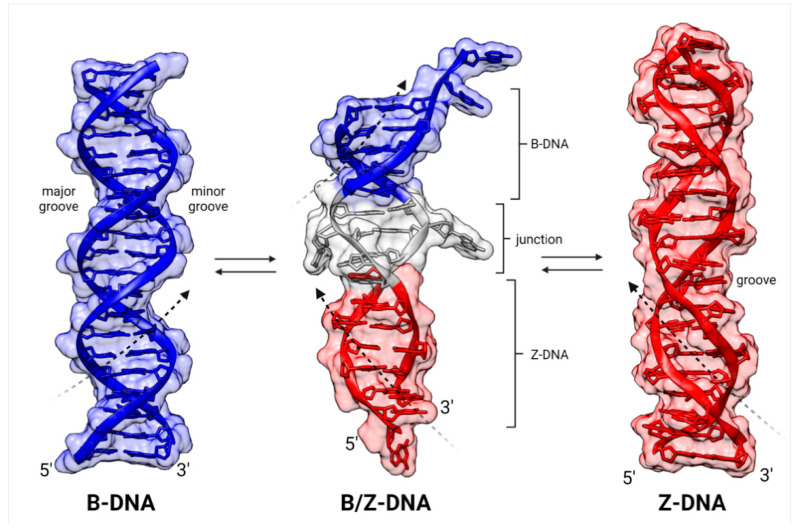
Comparative structures of alternative conformations of dsDNA: **left**, B-DNA; **right**, Z-DNA; **center**, a B-Z composite DNA with an intervening junction. Black dashed arrows indicate handedness (B-DNA, **right**; Z-DNA, **left**). Horizontal arrows indicate transitions between depicted dsDNA conformations. The figure was constructed with UCSF Chimera: B-DNA and Z-DNA are modeled structures, and B/Z-DNA is a crystal structure (PDB: 5zup). The helical pitch of B-DNA is 33 nm (10.5 bp/turn), and for Z-DNA, it is 46 nm (12 bp/turn).

**Figure 2:**
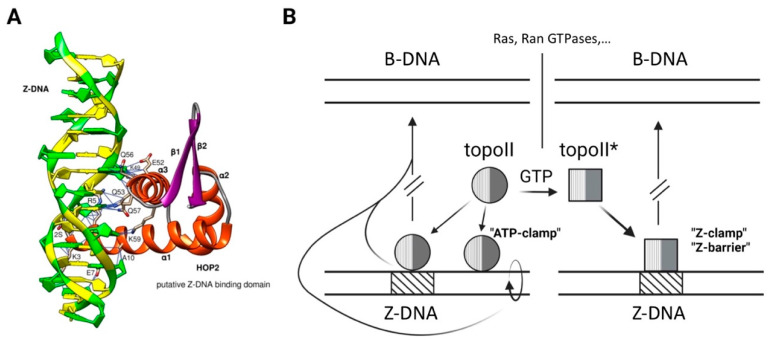
Prior bioinformatic and biochemical modeling instigating the search for a Z-DNA recognition domain of topoII. (**A**) Representative molecular docking of human HOP2 Zα region (aa 1–74) to Z-DNA [26]. Potential key amino acid interactions are depicted by thin blue lines. (**B**) Example of topoII as an allosteric ZBP (left and right). It is subject to competition by molecules (middle) exerting isomerase activity on nsc B-DNA segments. The relaxation process (ellipse with arrow, long curved line) abrogates the Z-conformation (short curved line) in the designated topologically linked segments. The affinity of topoII for Z-DNA is much greater than for B-DNA and increases further in the presence of GTP (topoII*), which also inhibits isomerase function (property 12, Table 1). These binding sites are deemed to constitute potential clamps, barriers, and crosslinkers, for example, in chromatin remodeling and mitosis/meiosis. Adapted from Figure 9 of Ref. [8].

In the cell biological context, DNA and RNA can adopt and maintain the left-handed Z-conformation, but usually only when stabilized by one or more Z-DNA- or Z-RNA-binding domains (ZBDs). Excluding antibodies, the RNA A-to-I-editing adenosine deaminase, ADAR1, was the second (see below) protein reported to strongly bind Z-DNA (not a substrate but a presumed targeting moiety [20]) and Z~RNA (the substrate) [21]). The editing function is key in the mediation of innate immunity directed against pathogens, e.g., RNA viruses, and endogenous retroviral elements. ADAR1 is also a major mediator of resistance to immunotherapies based on Immune Checkpoint Blockade (ICB) [22]. Zα, the winged helix-turn-helix (wHTH, WHD) DNA recognition domain of the interferon-induced ADAR1p150 isoform (residues 121–197) was identified in 1997 [21]. The crystal structure of its complex with a hexameric Z-DNA revealed key interactions with the characteristic zig-zag backbone and the pur-pyr alternation of glycosyl linkages and sugar puckering of Z-DNA [23] (Figure 1). Initial sequence searches based on Zα led to the identification of other host and pathogen proteins (“ZBPs”) exhibiting distinct, affine, functional interactions with Z-DNA and/or Z-RNA and likewise involved in host-pathogen response but also in stress response, cancer, autoimmunity, and germ cell DNA remodeling: ZBP1, PKZ, E3L, ORF112, RBP7910, ZBTB43 ([24] and references therein; [25]). In a recent study, we extended these findings by searching for new ZBPs with homologous Zα domains in the complete PDB structure database and in AlphaFold2 protein models [26]. A structure-based similarity search identified putative Zα ZBDs in 14 proteins with assigned structures and in 185 proteins modeled with AlphaFold2. STRING interaction networks revealed numerous functional clusters, one of which included HOP2 (with the highest Q score in the Zα domain search), a protein involved in stimulating strand exchange underlying homologous chromosome pairing in meiosis [27]. A HOP2-Z-DNA docking exercise led to the interaction image depicted in Figure 2A, in which the interaction is provided by α-helix 3 (α3) and supported by α-helix 1 (α1). Key amino acid interaction residues in this model are three glutamines (Q), three lysines (K), two glutamic acids (E), one arginine (R), one alanine (A), and one serine (S). A tyrosine residue (Y) believed to be crucial for Zα-Z-DNA interaction is located in β-sheet β1 but seems not to be directly involved in binding with our particular docking model. The Zα-ZBP family may be even more extensive; according to the current SMART non-redundant database (nrdb), there are 934 Zα domains in 478 Zα-protein homologs in various organisms [28]. The challenge is to establish their relevance, or the lack thereof, as functional ZBPs.

Following the advent of the Z-DNA crystallographic structures [9,10], extensive attempts were initiated (and persist) to define biological function(s) and identify specific protein interaction partners of left-handed dsDNA. In 1993, we reported the biochemical isolation, based on Z-DNA binding, of a ~165 kDa protein from *Drosophila melanogaster* cells and embryos with a 100-fold greater affinity for Z-DNA than for B-DNA [29]. It was unexpectedly yet unambiguously identified as the known cellular topoisomerase II (Top2; we will use the term topoII as generic for Type II, and particularly IIα, topoisomerases), a key member of the family of enzymes which resolve the inherent topological problems that arise with cellular nucleic acids [3,30]. TopoII was the first-reported non-antibody ZBP and exhibited a number of intriguing properties involving left-handed DNA, particularly the very high affinity and the pronounced allosteric influence of GTP. The latter was manifested by a significant enhancement of Z-DNA binding and the time-dependent emergence of enzyme inhibition and salt-resistant complexes (Table 1). Many properties were shared by the isoform topoisomerase IIβ and the topoII from other species, confirming that topoII primarily recognizes DNA secondary and/or tertiary structure rather than the primary sequence of DNA. Thus, the previously demonstrated higher affinity of the enzyme for bent or kinked DNA [31] was now extended to the non-B conformation Z-DNA, suggesting attractive models for accurately targeting the key topoII topology simplification activity to defined cellular loci. Unfortunately, the information of Table 1 languished until being revisited in a current retrospective account of left-handed DNA [8], incorporating an updated model for topoII function (Figure 2B). It was suggested that the techniques of bioinformatics and structural biology should be employed for elucidating the role(s) of topoII as a ZBP in the creation—as well as disassembly—of the DNA–protein complexes underlying topological (sub)domains. Such “topoclamps” could also serve as recognition targets and spatial delimiters of linear diffusion or “hopping” routes [32] for DNA-bound proteins, including topoII itself.

These results and the extensive in silico search for new Z-DNA/Z-RNA binding proteins mentioned above [26] led to the convergence of interests of the presenting labs with an initial focus on the verification of topoII as a ZBP candidate based on comprehensive Zα homology screening of the topoII family. This effort has revealed a novel, highly conserved “active zone” encompassing a winged-helix Zτ domain flanked by a GTP-binding site and a pervasive GxxG motif.

**Table 1:** Paralogous topoisomerases IIα (topoII) and IIβ are Z-DNA-binding proteins (ZBPs).

No.	Properties (1991–1994) ^a^	Ref.
	*Drosophila (D) topoisomerase II and human (H) topoisomerase IIα (topoII)*	
1	Two orders of magnitude higher binding affinity for Z-DNA than for B-DNA (D);	[29]
2	Complexes with Z-DNA salt resistant after 5 min;	[29]
3	Inhibition by linear Z-DNA of relaxation of co-incubated nsc minicircles (D);	[33]
4	Preferential affinity for and enhanced relaxation of ns (D) minicircles with Z-DNA forming insert (D, H);	[33]
5	Distinct DNA loci of binding and scission (cleavage/resealing);	[33]
6	VM-26 inhibitor-induced covalent DNA–protein complexes with minicircles ± Z-DNA forming insert (D);	[33]
7	Much greater affinity for intrinsically curved compared to linear B-DNA (D, H);	[34]
8	Hierarchy of DNA affinity: linear Z-DNA ≈ curved B-DNA ≥ nscDNA >> linear B-DNA (D, H);	[34]
9	No binding of ssDNA (D);	[29]
10	Increased formation of aggregates of nsc minicircles with Z-DNA forming inserts (D).	[33]
	*Effects of GTP or non-hydrolyzable GTPγS (much more effective)*	
11	Persistent, time-dependent, temperature-dependent inactivation of enzyme activity (D); incubation ± nscDNA;	[29]
12	Inhibition of DNA relaxation activity (D, H, calf thymus) via a proposed allosteric mechanism;	[29]
13	A 5–10 increase in affinity for Z-DNA and decreased affinity for B-DNA (D);	[29]
14	Inhibition of ATPase activity (D);	[34]
15	Relaxation inhibited by >4 mM ATP and >0.5 mM ITP but not by UTP or CTP;	[29]
16	Limited DNA compaction (knotting, catenation) by stoichiometric *Bombyx* and human topoII; GTPase activity ^b^.	[35]
	*human isoform topoisomerase IIβ*	
17	Hierarchy of DNA affinity: linear Z-DNA > nscDNA ≥ curved DNA >> poly[d(A-T)] > poly[d(G-C)].	[34]

^a^ Mg^2+^ always present. ^b^ a study not incorporating Z-DNA. nsc, negatively supercoiled.

## 2. Results and Discussion

### 2.1. TopoII Contains a Putative Z-DNA-Binding Domain (Zτ)

**Figure 3:**
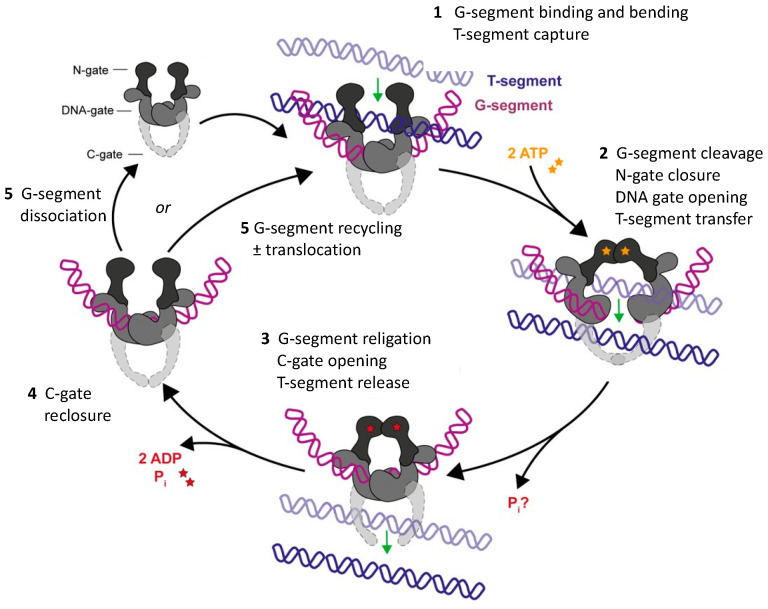
Canonical triple-gate isomerase mechanism for topoII. A double-helical DNA segment (G) binds to the topoII homodimer (upper left), is bent in the process, and is then cleaved, resulting in covalent protein (tyrosine)-DNA intermediates demarcating a double-strand break (DSB). A second “captured” DNA segment (T) traverses the DSB and is then released, while the G segment is religated, thereby restoring the integrity of the double helix. The intricate, sequential, concerted process is under allosteric control [48] mediated by ATP binding and turnover ([49], colored asterisks), divalent cations [50,51], and protein domains subject to post-translational modification, notably of the CTD [48]. The open and closed clamps of stages 1 and 3, respectively, are well depicted in a model of the tobacco enzyme (Figure 6 of Ref. [52]). Each cycle comprises a dual strand passage and thus changes the topological linking number *Lk* by ±2. The juxtaposition (a more appropriate term might be apposition) of the G and T segments at the crossover locus is dictated by the 3D structure of the local DNA domain, leading to numerous alternative topological outcomes [53]: resolution/simplification (relaxation, disentanglement) of plectonemic and toroidal supercoiled (+,−) substructures and reversal/formation of knots and catenanes arising during the processes of DNA transcription, replication, repair, recombination, higher-order chromosomal restructuring during mitosis and meiosis, and processing of closed circular DNA. Interference with DSB formation and resealing is highly genotoxic, and thus, steps 2 and 3 are key targets of antimicrobial and anticancer drugs [54,55,56,57,58]. Adapted from Figure 4 of Ref. [30].

**Figure 4:**
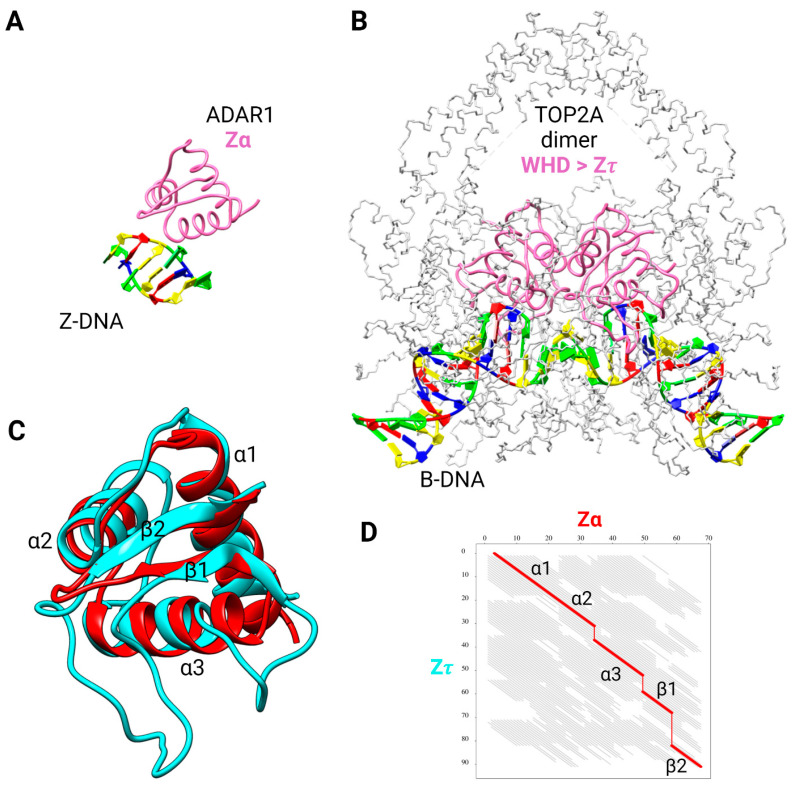
Structural comparison of Zα domain from human ADAR1p150 and putative Z𝜏 domain from human topoII. (**A**) Crystal structure of Zα in complex with d(CACGTG) (PDB: 3f21). (**B**) Cryo-EM structure of the human TOP2A DNA-binding/cleavage domain in State 1 (PDB: 6zy5). Zα or Z𝜏 domains are colored in hot pink, and DNA is colored according to NDB standards (A in red, T in blue, C in yellow, and G in green). (**C**) Superposition of Zα (ADAR1p150, red) and Z𝜏 (TOP2A 722–812, cyan) domains, canonical designation of helices and β-sheets is indicated. (**D**) Graph of FATCAT [60] chaining result intuitively showing structural similarity across all 91 aa-long alignment (thick red diagonals); three gaps are depicted using thin vertical red lines, and non-significant structural similarity is depicted by gray diagonals.

**Figure 5:**
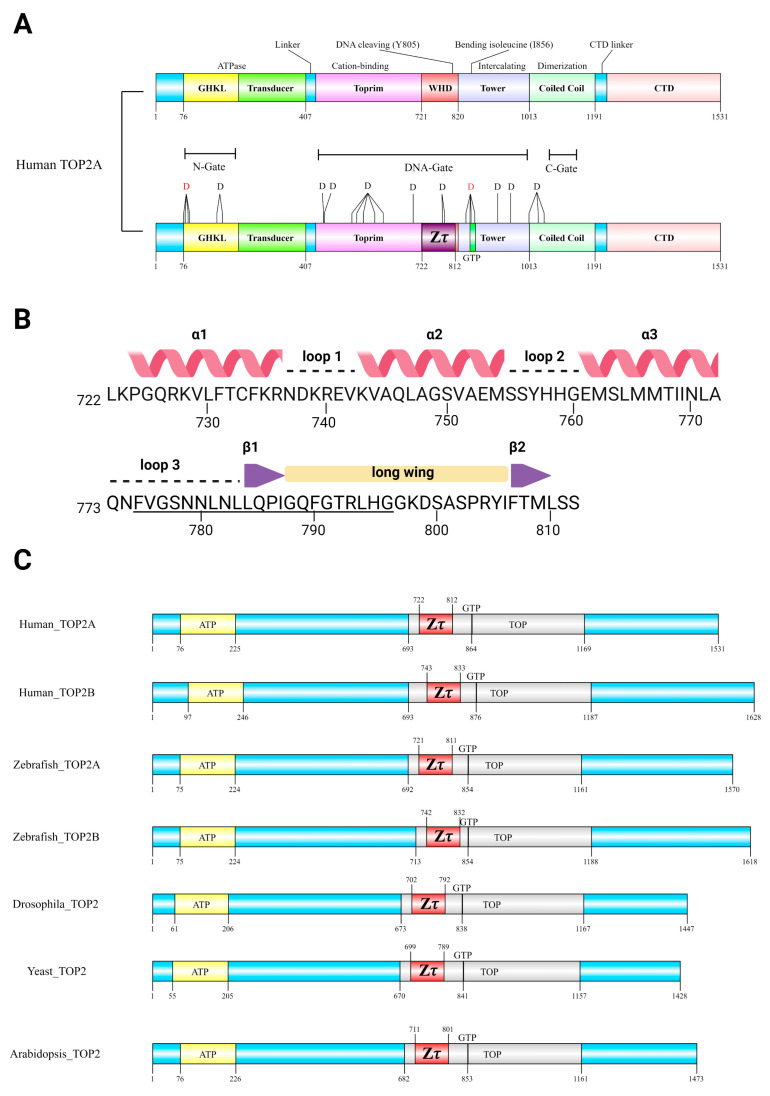
Domain composition of topoII together with putative Z-DNA-binding domains, Zτ (red), and predicted GTP-binding sites. ATP-binding sites (HATPase_c) and TOP (TOP4C) were annotated using a SMART web server [68]. (**A**) Known and newly identified features (Zτ, GTP-binding site, and deleterious SNPs, here depicted as Ds) in human TOP2A. (**B**) Zτ sequence (722–812), secondary structure, including the proposed “Z-discrimination region” (775–796, underlined residues; Table 3 below). (**C**) Evolutionary conservation of Zτ and GTP-binding sites in diverse eukaryotic species.

The two human paralogs TOP2A (170 kDa) and TOP2B (183 kDa) share ~70% sequence identity. TOP2A is expressed predominantly in proliferating cells, while TOP2B is present in all cells, including those in quiescent or differentiated states. Thus, TOP2A mediates DNA replication, chromosome condensation and decondensation, and sister chromatid segregation, whereas TOP2B is key in transcription and differentiation, particularly during neuronal development [36]. The fundamental linkage between DNA topological states and topoisomerase function [37,38,39] is reflected in the architecture of these enzymes (see Figure 5A below). The enzymatic core of both isoforms comprises three functional regions: the N-terminal N-gate/ATPase, the DNA-gate, and the C-gate. The isoforms also contain differing C-terminal domains (CTDs) that are largely unstructured and deemed to fulfil regulatory and targeting roles [40,41,42]. We briefly recapitulate the currently accepted [2,3,4,30,43,44,45,46,47,48] 3-gate (N, DNA, C) *isomerase* catalytic mechanism of topoII in the schematic representation of Figure 3. A molecular depiction of topoII is given in Figure 4B.

The vast majority of known protein sequences of Class IIA topoisomerases (>10^5^) are present in the domain Bacteria (88.5%), followed by the domain Eukaryota (7.2%), Archaea (2.0%), and Viruses (1.0%) (see Figure 7A below). The remainder of the protein sequences are still unclassified (1.3%). Interestingly, except for Viruses, the total number of protein sequences in each Domain of life is more than double the number of species, indicating that each species has more than two different types of Class II DNA topoisomerases on the average. This phenomenon most probably reflects the association of two separate protein subunits, as in bacterial gyrases (GyrA and GyrB) and the coexistence of topoisomerase IV (another member of the type IIA class) [42].

Based on our previous experimental findings and working hypotheses outlined in the Introduction, we searched for and identified a winged-helix domain, Z𝜏 (Figure 5A,B), in human topoII (TOP2A) that is structurally similar, albeit distinct, to Zα of ADAR1p150 (Figure 4). The WHD already identified in topoII (Figure 5A) overlaps Z𝜏 but is not strictly defined in the literature, being assigned to aa721—820 [59], to aa731—906 [45], or to an arbitrary region encompassing the active site catalytic tyrosine Y805. For the purposes of superposition, we selected particular regions of Zα and Z𝜏 and modeled them with AlphaFold to reconcile differences in the dozens of experimental structures available. The superposition of these (Figure 4C) revealed significant structural similarity with a *p*-value of 1.1·10^−4^ and root-mean-square deviation (RMSD) of atomic positions of 2.4 Å, based on 64 equivalent positions, and employing FATCAT flexible structural alignment [60] (Figure 4D). The slight discrepancies can be explained by the different lengths of the compared protein regions (represented by vertical lines in the figure): 67 aa for Zα from human ADAR1p150 and the significantly longer 91 aa for the Z𝜏 segment of human topoII. The long wing between the β1 and β2 sequences of Z𝜏 may confer greater affinity and specificity in its interaction with Z-DNA.

Table 2 quantifies the structural similarity of Zτ (from human topoII) to Zα of known Z-DNA/Z-RNA binding proteins (ADAR1, ZBP1, PKZ, E3, and ORF112) according to a number of parameters. In order to eliminate variations in different crystallographic approaches/quality of structures deposited in the RCSB database, AlphaFold models were always used since particular protein regions corresponding to Zτ or Zα/Zβ were predicted to have high, or even very high confidence. The highest number of aligned residues and the best RMSD and *p*-values scores are found in a pairwise comparison between Zτ (TOP2A) and Zα of human ADAR1p150. In contrast, sequence identity is very low (<10%) in all pairwise comparisons, suggesting that there is no detectable sequence homology. The *p*-value denotes the statistical significance of structural similarity. Human proteins Ubiquitin Fold Modifier 1 (UFM1, P61960) and histone H4 (P62805) served as negative controls.

All results, including the *p*-value, are much better for DNA-binding H4 than for UFM1, a membrane protein. Similar results were also obtained for Zτ from the human isoform TOP2B (Supplementary Material File S4), as expected in view of the high sequence similarity outside of the C-terminal domain (CTD). Only eight substitutions are present in the region corresponding to Zτ: L722F, S756A, M762Q, S763A, I769V, L781I, S800A, and S812T.

### 2.2. TopoII Contains a Major GTP-Binding Site

We next searched for significant GTP-binding sites in the human topoII, prompted by the results summarized in Table 1. For this purpose, we used the NSitePred tool [61], which was developed to accurately predict binding residues for ATP, ADP, AMP, GTP, and GDP via a sequence-based approach [60]. At first, we verified that the NSitePred tool is able to predict previously known and experimentally verified ATP-binding sites. We then directed our attention to GTP-binding sites. A very significant GTP-binding site (having the maximum score of 0.68 out of 1) was found in the TOP2A sequence at position I864, but the nearby sequence (852GAxGIxTGWxxKIPNF867) also showed GTP-binding potential. Worthy of note is that this region (particularly I856) is responsible for DNA bending, a key feature of the functional TOP2A dimer. Two isoleucines (one on each protomer) intercalate into the minor groove of DNA, bending the duplex by 130° [44]. A greatly reduced potential for GDP, ATP, and ADP binding at the same locus was predicted (Supplementary Material File S2). The results for topoII from diverse species including human are depicted in Figure 5C.

A second large cluster (149SNxDxxxxxVxxGRNGYGxKxCxxxxT175) of 13 GTP-binding sites was found in the region 149–175, spanning the known and experimentally validated ATP-binding region [62,63]. GTP was predicted to also bind strongly to this region (maximum binding score of 0.97), possibly even better than ATP (maximum binding score of 0.78; Supplementary Material File S2). These results suggest an even greater potential for strong allosteric control by GTP: promoting Z-DNA interactions (at the new GTP site) while concurrently inhibiting ATPase (property 14, Table 1) and thus isomerase (at the ATP sites). As a control of our calculations, the de novo predicted ATP-binding sites were in excellent congruence with the previously known sites. No GTP-binding site was found in human topoisomerase I (TOP1).

To put our data into further perspective, we extracted deleterious SNPs from the ENSEMBL Variant Database and filtered the most significant missense mutations using a strict threshold. There is a significant enrichment of these deleterious SNPs in the predicted GTP-binding locus and at the origin of the known ATP-binding domain, indicating a high functional relevance of these protein sites (red Ds in Figure 5A). There are also two such SNPs within the newly identified Zτ domain. Complete information about all 33 highly deleterious SNPs within human TOP2A is supplied in Supplementary Material File S1.

### 2.3. Both Zτ and GTP-Binding Site Are Phylogenetically Conserved across the Tree of Life

**Figure 6:**
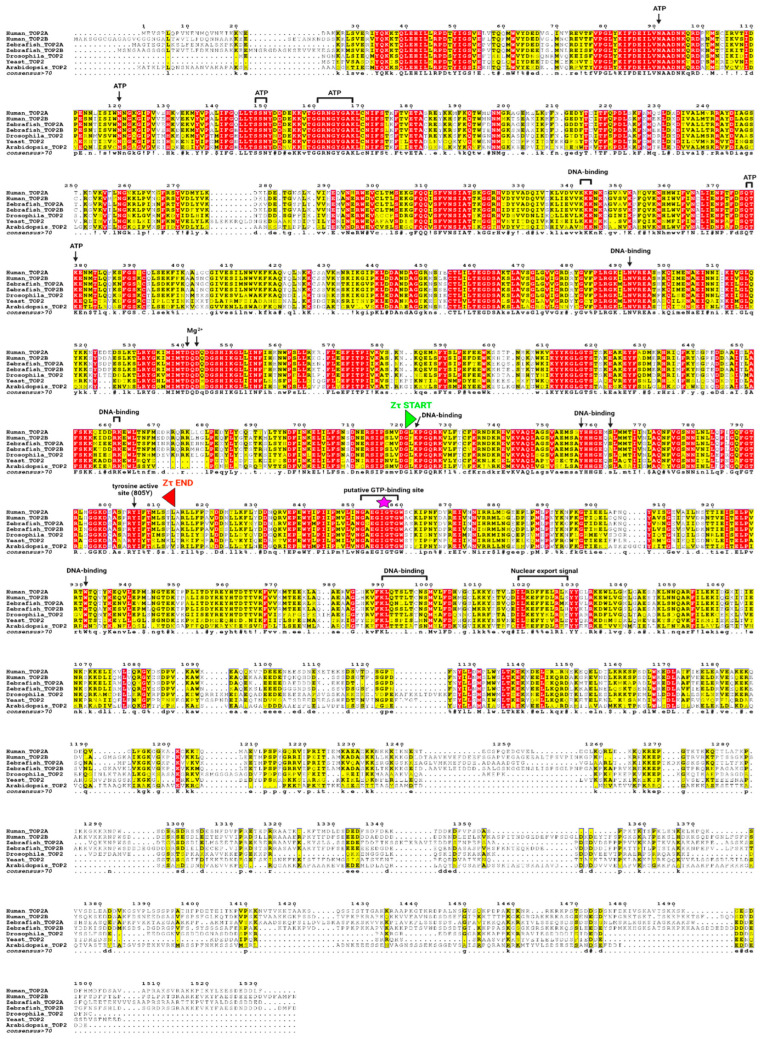
Multiple sequence alignment of TOP2 protein sequence in representative species showing very high conservation of the region Zτ constituting the Zα structural homolog (bounded by green and red triangle marks) and putative GTP-binding site. The purple asterisk mark highlights the locus critical for DNA bending. Experimentally validated ATP, Mg^2+^, and DNA-binding sites, together with the critical tyrosine 805 active site, are depicted as well. Columns highlighted in red and yellow show evolutionarily conserved regions/amino acid positions (primary sequence); the resulting consensus sequence is displayed in the bottom row, using criteria from MultAlin [69]: uppercase is identity, lowercase is consensus level > 0.5, ! is any-one of IV, $ is anyone of LM, % is anyone of FY, # is anyone of NDQE.

To depict the phylogenetical conservation of identified features (Zτ and putative GTP-binding site) in human TOP2A, we made a multiple sequence alignment of five representative eukaryotic species (Human, Zebrafish, Drosophila, Yeast, and Arabidopsis) (Figure 6). We then constructed a comprehensive multiple sequence alignment of all known metazoan topoisomerases of type IIA, particularly focused on their DNA-binding region (~400 aa). Nearly 1500 sequences were inspected and aligned to the Hidden Markov model logo (HMM) and about 350 sequences containing artifacts or truncated N or C ends were manually removed. In the rest of the sequences (1131), the putative Z-DNA-binding domain Zτ together with the newly identified GTP-binding region were most conserved.

**Figure 7:**
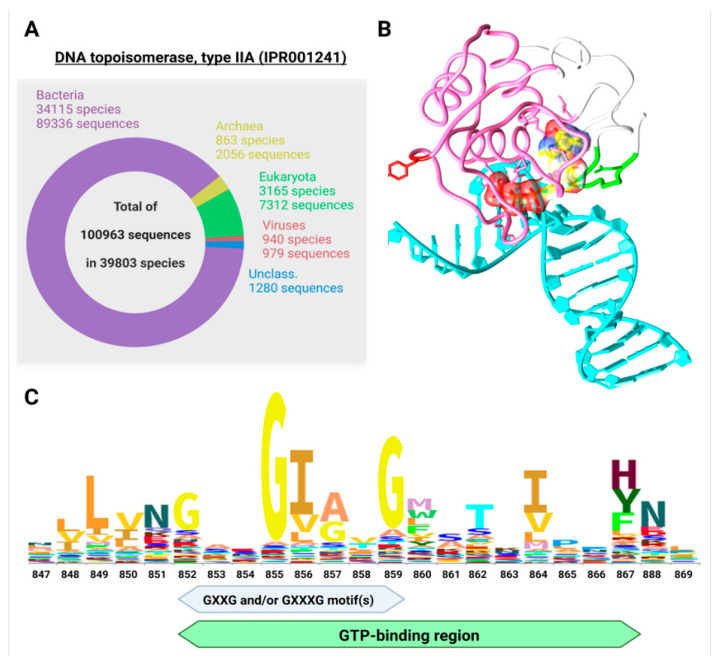
DNA Topoisomerase type IIA diversity (**A**) and GTP-binding (**B**,**C**). (**A**) Known diversity of Topoisomerase type IIA (IPR001241). According to InterPro Database, there are more than 10^5^ protein sequences in more than 50,000 different species across the whole tree of life. (**B**) GTP docked to the crystal structure of human topoII (PDB: 4fm9). Only the immediate surrounding of the docked GTP molecule is shown, but it comprises both the predicted GTP-binding region (in green) and Z𝜏. The tyrosine active site of TOP2A is in red. (**C**) Sequence logo of the region corresponding to the putative GTP-binding site, and the GxxG/GxxxG motif(s) based on seed alignment of the DNA topoisomerase IV (PF00521) domain. The logo was produced using the Skylign tool [71].

DNA topoisomerases of type IIA (TOP2A, InterPro Domain ID: IPR001241) are highly conserved in Metazoa [64] but can be found across the whole tree of life, as depicted in Figure 7A. In Bacteria, the related gyrase and TopoIV are also found (with some exceptions, such as the order *Corynebacteriales*). They have quite distinct properties and cellular functions [65]. In eukaryotic organisms, it is quite often that particular species contain several duplicated copies of TOP2A. Probably the most important event (from the anthropocentric point of view) occurred early in vertebrate evolution: the duplication leading to the paralog TOP2A and TOP2B genes [64,66]. A viral origin of eukaryotic topoisomerases was recently proposed [67].

### 2.4. Molecular Docking of Various DNA Types to Zτ

We further explored the possible GTP-binding potential of the human TOP2A protein by carrying out a computational docking procedure using a representative crystal structure of human TOP2A with bound DNA (PDB: 4fm9) [44] as a receptor, and GTP as a small ligand. One should note that the DNA in this case was in the B form, inasmuch as an experimental structure with bound Z-DNA is not yet available. Figure 7B indicates GTP docked very close to the predicted GTP-binding region, which also contains a highly conserved GxxG motif (Figure 7C), a key feature of K Homology (KH) domains and one which can provide local stereochemical flexibility [70].

**Figure 8:**
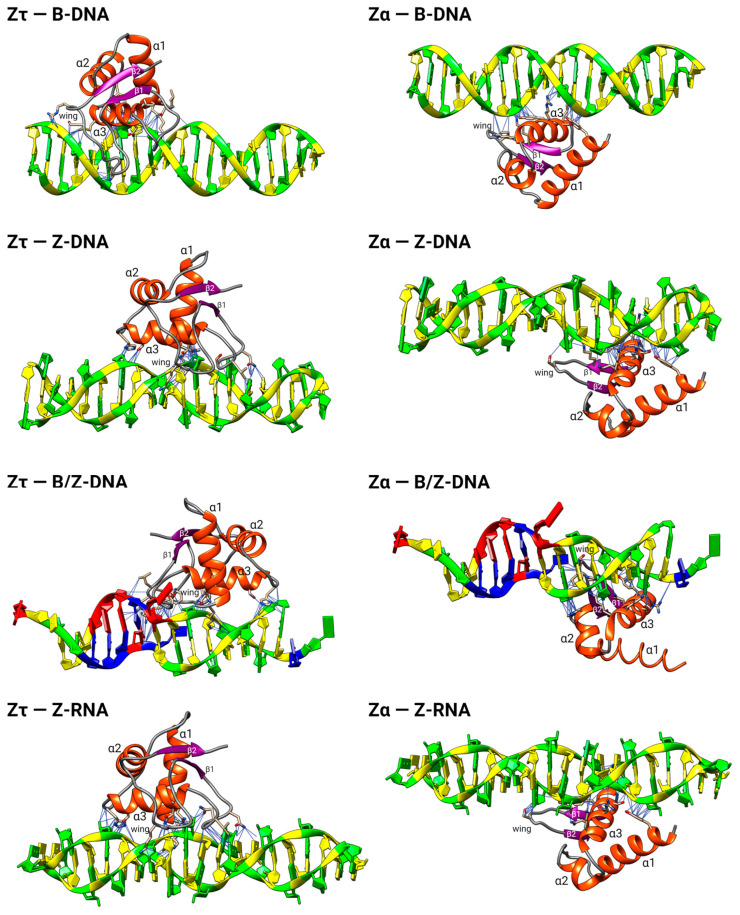
Molecular docking for various interacting protein–DNA pairs. The left column is for Zτ from the human protein TOP2A and the right column for Zα from the human protein ADAR1p150, both docked to the indicated nucleic acid structures (B-DNA, Z-DNA, B/Z-DNA, and Z-RNA).

We docked various nucleic acid structures (B-DNA, Z-DNA, Z-RNA, and B/Z-DNA) to the isolated Zτ domain of TOP2A (AlphaFold structure) (Figure 8). It appears that the Zτ domain of human TOP2A may interact with Z-DNA mainly through its α-helix α3 and β1-β2 loop, without the involvement of α-helices α1 and α2. Such results are illustrative yet inconclusive, inasmuch as the full extent of the protein–protein and protein–nucleic acid interactions of the homodimer is not represented. Fortunately, a control parallel exercise with the Zα domain (Table 3) reproduced the majority of residues and contacts denoted as idiosyncratic of the Zα family, based on extensive biophysical characterization [14,15,16,17,18,19]. The differences in the orientations of the two Z-domains relative to the DNAs are remarkable and await elucidation by high-resolution structure determinations.

**Table 3:** Parameters of protein–nucleic acid complexes obtained by molecular docking of Zτ from human TOP2A and Zα from human ADAR1p150 to left-handed Z-DNA and Z-RNA. Docking procedure: HDOCK. The docking score is calculated by a knowledge-based iterative scoring function; greater negativity usually implies a more feasible binding model. The confidence score empirically indicates the binding likeliness of two molecules (in the range of 0–1). Interacting aa residues in crystal structures 4fm9 (TOP2A in complex with B-DNA) and 3f21 (Zα in complex with Z-DNA) are shown as well. Shaded area: “Z-discrimination region”, common for all 3 docking interactions of Zτ with left-handed species (Figure 5B).

Zτ Docking Model					Zα Docking Model					Crystal	
Parameter	B-DNA	Z-DNA	B/Z-DNA	Z-RNA		B-DNA	Z-DNA	B/Z-DNA	Z-RNA	4fm9	3f21
docking score ^1^	−172	−175	−213	−208		−136	−179	−165	−193	-	-
confidence score ^2^	0.61	0.62	0.78	0.76		0.43	0.64	0.57	0.70	-	-
no. contacts	105	69	91	74		51	63	67	65	39	53
aa residue ^3^											
L722 *n*	1				Y136 *a*	1	2		1	K723	K169
K723 *b*	1, 2	1, 2	1	1, 2	H159 *b*		2			Y757	K170
Q726 *p*	2	1	2	2	K169 *b*	2		1, 2		H759	N173
Y757 *a*	2				K170 *b*		1	1	2	S763	R174
H759 *b*	2	2	1	1	N173 *p*		1	1	2	N770	Y177
G760 *n*			1	1	R174 *b*	1	1, 2	1	2	K798	T191
M762 *n*	1				Y177 *a*	2	1, 2	1	1, 2		P192
S763 *p*	2	2	1	1	S178 *p*	1	2		1		P193
T767 *p*	2				K181 *b*	1	2		1		
N770 *p*	2	1	1	2	K187 *b*	2	1		2		
L771 *n*	2				G190 *n*				2		
F775 *n*			1		T191 *p*		1	1 2			
V776 *n*		2	1	1	P192 *n*		2				
G777 *n*		1	1, 2	1, 2	P193 *n*			1			
S778 *p*		2	2								
N779 *p*			2	2							
N780 *p*		1, 2	1, 2	1, 2							
L781 *n*			2								
R793 *b*			1								
G796 *n*			1								
K798 *b*	1	1		2							
S802 *p*	1										
R804 *b*	1										
strand 1/(1 + 2)	6/14	6/12	10/17	7/14		4/7	6/13	7/9	4/10	1	1

^1^, more negative, better; ^2^, higher, better; ^3^, interaction with strands of nucleic acid double helix: 1, strand 1; 2, strand 2.; crystal structures, strand 1. Amino acid type: *a*, aromatic; *b*, basic; *n*, nonpolar; *p*, polar.

Interesting trends were observed for Zτ (Table 3). The best docking and confidence scores were obtained for Zτ with B/Z-DNA, followed by Z-RNA and Z-DNA. Zτ–B-DNA, a model of the canonical interaction of topoII with DNA, scored worse, a result compatible with the relative affinities for B- and Z-DNAs established for topoII (Table 1). Surprisingly, the docking model for B-DNA had 2.7× the number of interacting amino acid residues in the corresponding crystal structure 4fm9. The relatively high scores obtained with Zτ–Z-RNA raise the question as to whether class II topoisomerases (TOP2A, TOP2B) can bind productively to Z-RNA. In fact, topoII has been implicated in the regulation of viral replication [72], and most identified Z-RNA binding proteins to date have a role in (anti)viral mechanisms. GTP-binding proteins often engage in guanylate-mediated dimerization that endows them with antiviral properties [73].

In addition, Zα in the known ZBPs (ADAR1, DAI, ORF112, E3, PKZ) and the 14 best ZBPs we have predicted previously [26] are invariably located near the N- or C- terminus and are thus spatially exposed. Other regions of these proteins are not presumed to play a key role in the interaction with Z-DNA/Z-RNA.

In contrast, the Zτ of TOP2A occupies a central part of the protein, as does the DNA (G segment), such that other amino acid residues in the TOPRIM and TOWER domains are in close contact with the DNA [74]. For example, two tryptophan residues (W860 and W931) are involved in the crystal structure with bent B-DNA (4fm9). Tryptophan is a critical and well-described Z-DNA-binding residue in the Zα domain [18,20,23], and in 4fm9, W860 is in direct contact with the DNA backbone. In addition, it is at the center of the putative GTP-binding region predicted in this report. Interestingly, a conserved tryptophan in the core domain of rat transglutaminase (TGM2) is essential for catalytic activity [75]. TGM2 is a GTP-binding and hydrolyzing protein as well, interacting with topoII to promote DNA damage repair of DSBs in lung cancer cells [76].

### 2.5. Expanded “B-Z TopoII” Reaction Mechanism

The results and interpretations of the bioinformatics search featured above coupled with the prior biochemical data summarized in Table 1 constitute compelling evidence for the assertion that topoII possesses an inherent and pronounced affinity for left-handed Z-DNA. We also invoke below a putative capacity of topoII for catalyzing the right-to-left reversal in the helical sense of an attached DNA segment. If such an activity exists, topoII would represent a separate class of ZBPs, distinct from the family of proteins featuring the Zα domain and identified to date [14]. The implications regarding the functional repertoire of both partners (protein, nucleic acid) in the biological cell are profound.

**Figure 9:**
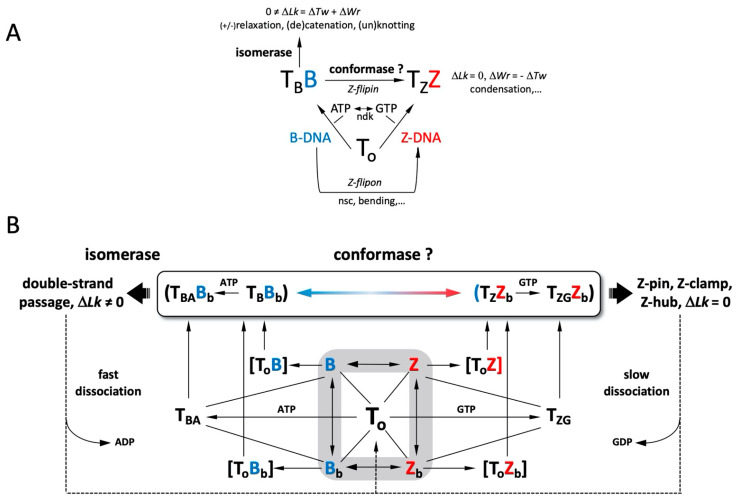
“B-Z-TopoII”: expanded reaction mechanism of topoII incorporating Z-DNA binding and a postulated conformase activity in addition to its canonical isomerase function. (**A**) Functional scheme, explained in the text. (**B**) Details of intermediate states (in square brackets) and outcomes of the isomerase and conformase pathways. The gray shaded area comprises the “DNA manifold” with interconversions between linear and bent conformations that depend on sequence, topological state, solution conditions, and external factors. Straight lines denote interactions between binding partners, leading to reactions (lines with arrows; in reversible reactions, a larger arrowhead indicates preferential state). Configurations of the topoII homodimer: **T_o_**, free; **T_B_**; bound bent (**B_b_**) B-DNA (G-segment); **T_Z_**; configuration bound to bent (**Z_b_**) (G-segment); **T_BA_**, **T_B_** with bound ATP; **T_ZG_**, **T_Z_** with bound GTP. Adapted from Figure 1 in Ref. [78].

The new mechanism (“B-Z TopoII” scheme) proposed for topoII is depicted in Figure 9 and summarized in Table 4**,** significantly expands its known repertoire as a topo*isomerase* (topo function 1, **tf1**) by incorporating three new features (**tf2**, **tf3**, **tf4**) into the standard topoII model of Figure 3. Acting in concert, these functions are deemed to fulfill essential requirements for maintenance of genomic DNA integrity and function: topological resolution, structural demarcation, and 3D (de)condensation and segregation. One should note that **tf1** targets 2 DNA segments, whereas **tf3** is considered to act on only one. In other words, the two activities “target” writhe (“writhase” or “crossover invertase”, [3]) and helical twist (“twistase”), respectively. ATP hydrolysis is essential for the catalytic function of **tf1** [77], but it is unclear whether it would also be required in **tf3**.

Panel A of Figure 9 provides an overview of the scheme, and panel B depicts certain features in greater detail. The apparent “B-Z” symmetry is more apparent than real, because the outcomes of the alternative pathways are quite distinct. In the “B-mode” of action, the topoII homodimer (**T_o_**) is shown to bind and process a B-DNA G-segment by adopting a quaternary configuration, **T_B_**, under allosteric control by ATP. The interaction with DNA leads to a complex, **T_B_B**, with two feasible fates, the first of which is to proceed through the isomerase cycle (Figure 3). The second fate arises if the proposed topo*conformase* activity (**tf3**) is manifested, such that **T_B_B** undergoes the transformation to **T_Z_Z**. In the alternative “Z-mode” of action, **T_o_** binds to a *preexistent* Z-DNA G-segment (see below), and the DNA gate (Figure 3) adopts an alternative configuration, **T_Z_**, in the stable complex **T_Z_Z** and does not proceed beyond stage 1 of Figure 3. **T_B_** incorporates the conformational mechanisms coordinating the inter-subunit interactions required for DNA cleavage [44]; **T_Z_** extends this notion to the quaternary structure favored for Z-DNA recognition.

GTP exerts a positive heterotropic allosteric influence on **T_Z_Z**, increasing its thermodynamic stability even more (property 13, Table 1). At the same time, it profoundly inhibits isomerase activity (properties 11, 12, 14, Table 1). **T_Z_Z_b_** ± GTP constitutes a highly stable topoII-Z-DNA end-state complex, with three possible consequences, neither of which leads to a change in the global ΔLk inasmuch as strand/helix passage is not involved. One eventuality is to clamp the distribution of supercoiled states within the topological domain encompassing the site of action. A second possible consequence is to act as a temporary, local storage site, maintaining the temporarily inactivated enzyme in nearby proximity for a subsequent required function. In this connection, it is relevant that the active site tyrosine lies *within* the Zτ segment of topoII, in contrast to the sequence-separated DNA-binding and catalytic elements of ADAR1p150. In the case of topoII, Z-DNA binding leads to enzymatic *inactivation*, whereas with ADAR1p150, Z-RNA recognition leads to *activation*.

TopoII possesses both ATPase [77] and GTPase (Figure 7 of Ref. [34]) activities. Considering the much greater efficacy of non-hydrolyzable GTP (Table 1), the scheme of Panel A presumes that the disruption of the **T_Z_Z** complexes occurs (albeit slowly, Figure 9B) upon hydrolysis of GTP. After the release of GDP, the protein reverts to its initial **T_o_** state and the DNA to its basal conformation dictated by the microenvironment. Finally, the **T_Z_Z** complex is deemed to serve as a recognition and/or structural element for establishing higher order single- or multicomponent complexes. There are two additional features in the scheme of panel A to note. One of them is the potential equilibration between local B- and Z-conformations in the *absence* of topoII, i.e., depending on factors such as DNA sequence, state of deformation from torsion (supercoiling), tension (stretching), bending, and solution parameters (temperature, salt composition, small effector molecules such as polyamines, ionic strength, dielectric constant). Such sequence domains that can fluctuate relatively easily between the left- and right-handed helical conformations constitute the class of ***Z-flipons*** introduced by Allan Herbert for DNA (and RNA) sequences that “flip” into the Z-conformation, are recognized by ZBPs [79] and thereby exert a number of cell biological functions [17,18,20]. For sequences that *require* the putative conformase function of topoII to adopt and stabilize a left-handed conformation (**fp3**, Table 4), we propose the new term ***Z-flipins***. The other noteworthy feature is the exchange reaction mediated by ubiquitous nucleoside diphosphate kinases (ndks), permitting the facile interconversion of ATP and GTP via their respective diphosphates.

Panel B of Figure 9 provides a more detailed view of “B-Z-TopoII”. It stresses the key role of a particular, essential feature of topoII isomerase function (Figure 3), the *bending* of the G-segment [80,81,82]. Such a “bind-then-bend” mechanism [83] is shared with many nucleic-acid-binding proteins [84,85,86], and bending has been newly demonstrated to constitute a physical means for promoting *by itself* the B-to-Z transition under physiological conditions [87]. The latter finding provided a major impetus for proposing a *conformase* capability of topoII. Pre-bent DNA is a preferred binding target for topoII ([31]; property 7, Table 1) and enzyme-induced flexibility is invoked as the means for selecting cleavage sites [88], but the claim of a potential conformase activity presumes an *obligatory* intervention by the protein in order for the deformation (bending)-facilitated B-Z transition to occur.

In the absence of direct structural data, we can only speculate as to whether the entire length of the bound G-segment would adopt the Z-conformation in a concerted reaction [7] or whether a sequential transformation, such as BjBjB → BjZjB → ZjZjZ → Z (j, unpaired junction) would be more likely, perhaps even involving the Z(WC)-DNA alternative left-handed double helix proposed to overcome the inherent “chain-sense paradox” of crystallographic Z-DNA [89,90]. It is also conceivable that the isomerase pre-scission intermediate exhibiting the A-DNA conformation within the G-segment DNA [80] may participate in a BjBjB → BjAjB → BjZjB → ZjZjZ → Z conformase reaction sequence. A- and Z-DNA share certain features: dependencies on hydration state and counter-cations, water bridging of free phosphate oxygens, and purine sugar pucker, and the B, A, Z interconversion landscape is exceedingly complex (90], Figure 2 of Ref. [91]). An intriguing question is whether the existence of an obligatory A-conformation intermediate in the isomerase cycle implies a B → A “conformase” property for this core function (**tf1**) of topoII as well as B → Z in **tf3**.

The other currently known class of Zα based ZBPs share many structural and functional properties [14,15,16,17,18,19], but these do not include DNA bending. Z-DNA “inducibility” is also ascribed to some of these ZBPs, yet in our estimation, unambiguous experimental demonstration is lacking for a *catalytic* activity (with turnover), instead of, or in addition to, the selective binding preference for the left-handed conformation. This issue arose early in Z-DNA research in relation to anti-Z-DNA antibodies, but can and has been resolved kinetically (Figure 10 and Figure 11 of Ref. [92]).

In the isomerase pathway, the “cleavage-competent” bending of the G-segment DNA (step 2, Figure 8) [51] is accomplished by Mg^2+^ coordination to the TOPRIM domain [93] without involvement of direct amino acid side chain-base contacts [80]. Instead, a topoII-invariant isoleucine intercalates into and widens the minor groove, thereby increasing DNA rise and roll while decreasing twist and, thus, the charge density of the helix [94], effects which by themselves would also favor the B-to-Z transition and thereby enable the alternative conformase pathway. This “local conformational micropolymorphism” [95] provides the flexibility required to achieve a bend of ~120–150° [80,96]. Furthermore, in vitro studies have demonstrated that the application of moderate tension (stretching) greatly reduces the requirement for torsional stress (untwisting) in the B-Z transition induced by supercoiling [97]. Structurally, the finding that GTP binding dramatically increases the affinity for Z-DNA while decreasing the affinity for B-DNA (Table 1) implies that the quaternary conformations **T_Z_** and **T_ZG_,** as well as **T_B_** and **T_BA_,** differ in significant ways, and DNA stretching (longitudinal tension) may well be involved. More generally, it appears that topoII is representative of proteins that engage nucleic acids in a manner that exploits the capacity of *both* macromolecules to undergo mutual conformational adaptations that provide thermodynamic stability and specific recognition via enthalpic-entropic compensation [94,98,99]. In the case of the DNA, the nucleotide sequence is a (the) major factor. Yet, in a real way, the conformase activity that we are invoking for topoII encompasses *both* the protein and DNA, which is to say that “the enzyme and the substrate are one” (the biochemical equivalent of “it takes two to tango”). CRISPR-Cas9 is a prominent albeit complex example of such concerted conformational adaptation. Protein recognition and catalytic activation ensue upon an open-to-closed domain rearrangement in concert with DNA twisting, bending, and base flipping, all pursuant to initial successful pairing of the guide RNA and R-loop formation [100]. An intriguing Z-to-B-DNA remodeling protein (ZBTB43) has also been reported [25].

Another notable feature of the Z-mode function depicted in Figure 9 is its temporal behavior. Simulations of the system originating from B-DNA and the **T_o_** state can exhibit damped oscillatory responses culminating in steady-state levels of free and protein-associated Z-DNA and topoII-Z-DNA complexes. The system may thus comprise an inherent memory” property, distinct yet related to the rationalizations of the capacity of topoII to generate topological distributions “beyond thermodynamic equilibrium” [101]. In the latter case, the inherent supercoil-dictated directionality of the DNA-gating mechanisms (Figure 8, [2,45]) and the perturbed counterion distributions of juxtaposed helices [94] undoubtedly contribute. However, in the Z-mode of topoII, the longevity of exposed (B)-Z-(B) segments would be a primary factor, and they may account for the hysteretic behavior reported for complexes of anti-Z-DNA antibody with supercoiled ccDNA carrying Z-forming inserts [102].

### 2.6. A Case Study of the “B-Z TopoII” Mechanism: Mitosis

We now test the applicability of the “B-Z TopoII” mechanism to rationalize aspects of cellular mitosis, the autopoietic [103] process underlying cell division in which the expression of topoII peaks (at G2-M, there are ~10^6^ molecules/cell [104]) as it executes the essential functions of chromosomal DNA condensation and then segregation [46,105,106], distinct from its contributions to genome stability and organization in interphase [3]. The sequential progressive stages of the mitotic cell cycle (G2-interphase → prophase → prometaphase → metaphase → anaphase + telophase → cytokinesis) are precisely choreographed [105,106,107,108,109,110,111,112] and are accessible to high-resolution microscopy [105]. In prophase, the topologically associated domains (TADs) of interphase are disrupted, and the 6.3 Gbps (human diploid) DNA is organized by condensin II in a process of loop extrusion (LE) into ~4·10^4^ loops of ~450 kb. These are fixated at their base by dynamic [113] ring-shaped protein complexes (SMC, structural maintenance of chromosomes) aligned so as to form the axes of the sister chromatids. In prometaphase (and again in anaphase), the primary condensin II loops are further partitioned, 5–10-fold, by condensin I association into ~2·10^5^ nested ~90 kb off-axis subloops (the ~1 topoII/condensin-1-loop stoichiometry is intriguing), resulting in pronounced DNA overwinding and progressive compaction. The latter continues and achieves a maximum, ~10^4^-fold, *after* sister chromatid separation in anaphase [105,114].

In metaphase, TopoII is the most abundant protein component of the chromosome scaffold, followed by condensins I and II and chromokinesin KIF4 [106,107]. TopoII is bound to the chromosome axes and centromeres and is a key and indispensable participant in the processes outlined in the preceding paragraph [46,115,116]. Particular emphasis has been placed on the interplay between the respective roles of topoII and condensin II/KIF4 [112]. This focus arises because the fundamental activities of the two components (LE/compaction vs. topological simplification) are seemingly antagonistic in the coordination of DNA condensation with the equally requisite and concurrent elimination of spurious knots, tangles and sister chromatid interlinks [115]. Lateral chromosomal compaction has been attributed to condensin and KIF4 and axial compression to topoII [117]. Adding to the complexity of the system are: a dual driver–damper role of two condensin ATPases [108]; the intervention of other topoisomerases, chromokinesins, cohesin, and cyclins; and extensive temporally synchronized protein modifications [118], notably (de)phosphorylation and sumoylation, such as of the DNA-gate and the C-terminal domain (CTD) of topoII [41,112]. The mitosis literature abounds with conundrums, assertions, and still open questions, including the following:

- What are the ultrastable topoII-DNA complexes that play a structural role in chromosome architecture? [43]
- Do centromeres *drive* chromosome compaction?
- How do non-B-DNA centromere sequences participate to (de)condensation?
- How does topoII contribute to axial shortening of the chromosomes [117]?
- How is cohesin release coordinated spatiotemporally with the actions of condensin and topoII in sister chromatid resolution [119]?
- What are the kinetic pathways of topology simplification in metaphase chromosomes [47,120]?
- How is *large-scale* compaction and spatial arrangement achieved [109]?

A detailed treatment of the above is beyond the scope of this publication. Yet, we can invoke features of the “B-Z-TopoII” mechanism of Figure 9 to address some of the issues. We start by noting that evidence exists for localized protein interactions with genomic DNA, including: the recruitment of topoII to SAR/MAR (nuclear scaffold/matrix attachment) sites [43], which can assume a variety of non-B-DNA conformations [121]; the interactions of flipons and nucleosomes [122]; and 40 years of chromosome immunochemistry with anti-Z antibodies, revealing localized binding to heterochromatin. At this juncture, we propose the following scenarios incorporating B-Z-TopoII in mitosis as worthy targets for experimental verification. The aim is to specify a robust mechanism, one applicable to all chromosomes and organism expressing a topoII, and mindful of Ref. [106]: “Our data point to a role for TOP2A as a structural chromosome maintenance enzyme locking in condensation states once adequate compaction is achieved”.

GTP fulfills multiple functions in the cell: nucleic acid precursor, energy source, and messenger/allosteric regulator of protein synthesis, cytoskeleton dynamics, intracellular transport, signaling, and organelle function [123]. In contrast, ATP is utilized as a (the) general cellular energy carrier and phosphoryl donor. The mean cellular GTP concentration is ~1/10th that of ATP (<1 mM, >1 mM, respectively [124]); both are under tight metabolic regulation [103]. However, the synthesis of GTP is compartmentalized, leading to the notion that its production—by nucleotide salvage, de novo biosynthesis, and nucleoside diphosphate kinase activity—and consumption may generate gradients that affect cellular phenotypes in accordance to the immediate spatiotemporal demands of the cell [123]. The metaphase–anaphase stages of mitosis are such a case because chromosome segregation requires sister kinetochores at the centromere to attach microtubules emanating from opposite spindle poles. The small GTPase, Ran-GTP, promotes spindle assembly around chromosomes [125,126] by locally delivering cargoes (importin-bound spindle assembly factors, SAFs) that regulate microtubule dynamics and organization [127]. Because RCC1, the RanGEF (Ran guanine nucleotide exchange factor), is chromatin associated, a strong negative gradient of activated Ran-GTP is established, radiating from the kinetochores to the spindle poles [126,127]. Growing microtubules, associated motor proteins, and Ran-GTP require GTP hydrolysis for function. The local levels of GTP must be accordingly high.

We recall from Table 1 (properties 7, 8) that topoII exhibits a graded affinity for non-canonical DNAs and now postulate that high prometaphase levels of GTP at the centromere convert a substantial fraction of the resident topoII molecules to the **T_ZG_** species via function **tf4** (Table 4). These lead to chromosomal compaction at the centromeres, where topoII and preexistent and/or potential Z-form segments of α-satellite DNA are concentrated [128,129,130,131]. This process occurs via (a) function **tf3** (binding to flipons, e.g., at the base of condensin-1,2 loops); and (b) function **tf2** at suitable loci (e.g., flipins at loop apices), given appropriate conditions of DNA helical bending, tension, torsion, and sequence. Isomerase function **tf1** is inoperative except at positions of high topoII occupancy [132], where limited GTP-driven rounds of catenation and knotting, both contributing to compaction, can occur (property 16, Table 1). The dimerization capacity of certain GTP proteins [73] alluded to earlier implies that topo-topo crosslinks, as well as Z*-DNA, a self-associated form of Z-DNA [8], may contribute to the axial compression evident during and after metaphase. Topological “redistribution” is also a key factor in normal compaction (from the Abstract to Ref. [133]: “The results suggest that the local deformation caused by protein binding can yield a global configurational change, dominated by slithering, which brings two (originally) remote sites to close proximity, and that the nature of such effect is related to the sequence architecture.”). The great affinity of topoII for Z-DNA would confer a temporal stability in the metaphase stage, and it is perhaps indicated by the fractional non-recoverable population in FRAP determinations performed on mitotic chromosomes [40]. As in the case of the inhibitor, etoposide [53], loop trapping at Z-clamps may block sliding of topoII on the DNA, thereby increasing its action as a roadblock.

Upon exit from metaphase, the Ran-GTP gradient and high GTP concentration dissipate, and cohesin is released from the centromeres, unlocking the sister chromatids [116,134,135]. Flipins revert to the B-conformation as topoII is released, regaining the **To** conformation with isomerase (**tf1**) functionality. It can thus proceed to decatenate and unknot residual inter-chromosomal links, insuring error-free segregation. The chirality dependence of human topoII dynamics (+ over − supercoiled DNA) may also be a factor [53].

Is there any evidence for the mechanisms proposed above? Mutants with phenotypes indicative of selective inactivation of the individual functions of Table 4 would be relevant. The literature is indeed replete with mutations of topoII, particularly in reference to topo inhibitors/”poisons”, and their distinctive influence on isomerase function and processivity [136]. However, it is difficult to conceive of unambiguous selection strategies for isomerase+/Z-binding- mutants in view of the functional overlaps envisioned in the proposed “B-Z topoII” scheme. Nonetheless, such a phenotype may apply in the case of a reported allele, *top-2(it7ts)*, of TOP-2, the single topoisomerase II homolog in *C. elegans* [137]. An arginine → cysteine (R → C) missense mutation at residue 828 (corresponding to residue 793 of huTOPIIα) leads to failure of segregation during anaphase I of meiosis, resulting in anucleate sperm. The segregation defects are not due to residual entanglements incurred during meiotic DNA replication, implying a possible **tf1**+/**tf2(tf3,tf4)**- (Table 4) status of topoII. The authors write: “We propose that TOP-2 localization during late pachytene positions the protein to function in chromosome condensation/karyosome formation prior to the meiotic divisions. When TOP-2 localization is disrupted in the *top-2(it7ts)* mutant, either abnormal or insufficient chromatin remodeling occurs during late prophase resulting in aberrant chromosome segregation”. A second publication [138] deals with the sensitivity of wild strains of *C. elegans* to etoposide poisons depending upon whether they carry a methionine → glutamine (M → Q) substitution in TOP-2, residue 797. The non-polar methionine increases hydrophobic interactions between the protein and the etoposide, resulting in increased genomic instability. Residue 797 is conserved from yeast to humans but exhibits one of the few differences between the two human topoII isoforms (huTOPIIα M762, huTOPIIβ Q778). HuTOPIIα M762 and R793, featured in the two cited publications are identified as DNA interactors in our Zτ docking model (Table 3). R793 is located in what we have designated as the “Z-discrimination region” such that the charge altering R → C substitution would be very significant.

We conclude that while the above discussion of mitosis does not incorporate the complex interplay of myriad other proteins, including histones, and their programmed modifications, it provides a plausible cellular context for the B-Z-TopoII scheme of Figure 9. The potential for extending the concepts to detailed treatments of meiosis and interphase chromatin is obvious.

### 2.7. Perspectives and Biomedical Outlook

This publication offers new paradigms for the biological relevance of left-handed double-stranded DNA (RNA?) and for the functions of Type II (and possibly other) topoisomerases. Confirmation, elaboration, and extension will require substantial efforts in the fields of molecular, cellular, and structural biology, including ultrahigh-resolution imaging [139] but also in medical pharmacology. The Z-DNA related properties of topoII described in this publication, potentially shared with some of its interactome partners [140], offer the prospect of new antiproliferative compounds, pharmacologically complementary to the numerous existing anticancer drugs targeting the isomerase mechanism of the protein. Current strategies for topoII-based drug discovery [56,57,58,141,142] lend themselves to this goal. GTP-binding site-specific compounds based on non-hydrolyzable nucleotides, isomerase inhibitory purine scaffolds [143] or suitably adapted ATP-competitors [144] would introduce a new dimension of target selectivity. Small molecule Z-DNA interactors or inducers [22] are of potential interest as well. Combination therapy also lends itself to physical means for precision targeting minimizing off-target toxicity, for example, by exploiting superparamagnetic nanocarriers, click chemistry, and magnetic focusing.

## 3. Materials and Methods

### 3.1. Structural Similarity Analysis of Human Z𝜏 (TOP2A) and Various Proteins Containing Zα Domains

As a representative example of Z𝜏 (from human TOP2A) and Zα (from human ADAR1) structural similarity (Figure 3), the following structures were used: Cryo-EM structure of human topoisomerase IIα DNA-binding/cleavage domain in State 1 (PDB: 6zy5) [48], and crystal structure of Zα in complex with d(CACGTG) (PDB: 3f21) [145]. The structural similarity for statistical comparison (Table 2) was computed using the FATCAT approach [60] (accessed from https://fatcat.godziklab.org/fatcat/fatcat_pair.html, 25 February 2023) with the flexible alignment mode. AlphaFold-modelled [146] PDBs obtained from the UniProt database [147], i.e., human ADAR1p150 (P55265), human ZBP1 (Q9H171), PKZ from *Danio rerio* (Q5NE12), E3 from *Vaccinia virus* (P21605), and ORF112 from *Cyprinid herpesvirus 3* (A4FTK7), and negative controls UFM1 (P61960) and H4 (P62805) from *Homo sapiens*. Only regions corresponding to particular Z𝜏 and Zα domains were always used as input (Supplementary Material File S3). To visually show the structural similarity of particular regions, UCSF Chimera molecular modeling system [148] and toolkit [149] were used.

### 3.2. Searching for Putative GTP-Binding Sites within Topoisomerases

Putative GTP-binding sites (together with ATP-, ADP-, AMP-, and GDP-binding sites) within protein sequences of interest were predicted using a Nsitepred web server [61] (accessed from http://biomine.cs.vcu.edu/servers/NsitePred/, 2 January 2023). This tool computes the so-called binding probability for both GTP/GDP/ATP/ADP/AMP (on a scale of 0–1) for each amino acid residue. Default parameters were used, and raw results were obtained in tabular format and further processed/filtered/described in Microsoft Excel (these processed data are available as Supplementary Material File S2).

### 3.3. Searching for Deleterious SNPs within Human TOP2A

Deleterious SNPs with missense consequence in the human *TOP2A* gene were extracted from the ENSEMBL Variation resources [150] accessed from https://www.ensembl.org/Homo_sapiens/Gene/Variation_Gene/Table?db=core;g=ENSG00000131747;r=17:40388525-40417896, 9 March 2023. Strict filtering criteria were used: SIFT [151] score ≤ 0.05; PolyPhen [152] ≥0.95; REVEL [153] ≥0.65; and Mutation Assessor [154] score ≥ 0.9. Only SNPs meeting all criteria at the same time were chosen as highly deleterious ones.

### 3.4. Multiple Sequence Alignment of Full-Length TOP2 Protein Sequences

Seven representative and phylogenetically diverse protein sequences of Topo II, including Human TOP2A (UniProt Protein ID: P11388), Human TOP2B (UniProt Protein ID: Q02880), Zebrafish TOP2A (UniProt Protein ID: Q5PQY4), Zebrafish TOP2B (UniProt Protein ID: Q1LUT2), Drosophila Top2 (UniProt Protein ID: P15348), Yeast TOP2 (UniProt Protein ID: P06786), and Arabidopsis TOP2 (UniProt Protein ID: P30182), were used to construct multiple sequence alignment within UGENE standalone tool [155]. The following parameters were used: mode MUSCLE default, max iterations = 3, -stable (do not rearrange sequences). ESPript 3.0 tool [156] (accessed from https://espript.ibcp.fr/ESPript/ESPript/, 1 January 2023) was used for alignment rendering and producing figures for publication. The following sequence similarities depiction parameters were used: Similarity coloring scheme—%Equivalent (a percentage of equivalent residues was calculated considering physico-chemical properties); Global score = 0.7; Display consensus seq: -yes. Alignment color scheme: -flashy; the number of columns: 140. Additional functional features (active sites, etc.) were added manually using Biorender, according to UniProt [147] features (accessed from https://www.uniprot.org/uniprotkb/P11388/entry, 2 January 2023).

### 3.5. Molecular Docking

Molecular docking was performed using the HDOCK tool [157] (accessed from http://hdock.phys.hust.edu.cn/, 27 January 2023). The following structures were used as an input: AlphaFold structure of Zα domain of human protein ADAR1 (AF-P55265-F1, region corresponding to amino acid residues 133–199); AlphaFold structure of putative Z𝜏 domain of Topo II (AF-P11388-F1v2, region corresponding to amino acid residues 722–812); CG dodecamer in B-DNA form modeled on x3DNA-DSSR webserver (accessed from https://x3dna.org/, 5 January 2023) [158]; CG dodecamer in Z-DNA or Z-RNA forms modeled using 3D-NuS webserver (accessed from https://iith.ac.in/3dnus/, 3 January 2023) [159]; and crystal structure of B-Z junction obtained from RCSB PDB database (accessed from https://www.rcsb.org/structure/5zup, accessed on 7 January 2023) [160]. As a “Receptor molecule”, protein structures were used, and the structures of nucleic acids were always designated as “Ligand molecule”. Default parameters for docking procedures were used, except for our choice to use template-free docking only. Obtained models were then visualized in UCSF Chimera [148] and are enclosed in pdb formats in Supplementary Material File S5. GTP molecule (obtained from https://pubchem.ncbi.nlm.nih.gov/compound/guanosine-triphosphate, 2 February 2023) was docked to the structure of human TOP2A (PDB: 4fm9) using the PATCHDOCK web server for rigid docking with default parameters [161], and the resulting model is also enclosed in pdb format as Supplementary Material File S6.

## Figures and Tables

**Table 2:** Structural parameters of similarity between Zτ of human topoII (P11388) and Zα/Zβ of various proteins: human ADAR1p150 (P55265), human ZBP1 (Q9H171), PKZ from *Danio rerio* (Q5NE12), E3 from *Vaccinia virus* (P21605), and ORF112 from *Cyprinid herpesvirus 3* (A4FTK7), together with negative controls (n.c.) UFM1 (P61960) and H4 (P62805) from *Homo sapiens. p*-values lower than the 0.05 threshold are in italics.

Feature	Parameter	Units	Domain								
			Zα	Zβ	Zα1	Zα2	Zα	Zα	Zα	n.c.	n.c.
protein			ADAR1p150		ZBP1		PKZ	E3	ORF112	UFM1	H4
RMSD	structure	Å	2.4	2.7	2.7	3.0	2.6	2.6	2.7	3.2	2.8
*p*-value	structure	10^−3^	*0.11*	*1.3*	*0.39*	*2.8*	*0.24*	*0.34*	*0.70*	200	55
equivalent positions	structure	no.	64	62	60	61	60	61	60	39	50
gaps	sequence	%	30	32	34	33	34	32	34	39	7
identity	sequence	%	7.7	8.8	3.3	5.5	2.2	3.3	6.6	9.4	7.4
similarity	sequence	%	26	24	17	18	18	19	22	19	22

**Table 4:** Expanded functionality of topoII (“B-Z topoII”). The question marks indicate potential RNA targets that have yet to be investigated.

Function	Activity	Target(s)
**tf1**	*isomerase*: double-helix passage (Δ*Lk*)	B-DNA
**tf2**	high affinity recognition and stabilization of left-handed double helix; no covalent protein–DNA intermediate	Z-DNA, Z-RNA?
**tf3**	*conformase*: induction of the right-to-left transition in double-helical sense	B-DNA, A-RNA?
**tf4**	pronounced positive heterotropic allosteric role of GTP in **tf2** and **tf3**	topoII

## Data Availability

All data are available in the manuscript and within Supplementary Materials.

## Supplementary Materials

The following supporting information can be downloaded at: https://www.mdpi.com/article/10.3390/ijms241310740/s1.
