# Supplementary material for: Extensive Bioinformatics Analyses Reveal a Phylogenetically Conserved Winged Helix (WH) Domain (Zτ) of Topoisomerase IIα, Elucidating Its Very High Affinity for Left-Handed Z-DNA and Suggesting Novel Putative Functions
Source: Int J Mol Sci. 2023 Jun 27;24(13):10740. doi: 10.3390/ijms241310740 (PMC10341724; doi:10.3390/ijms241310740)
Supplement: Supplementary file 1 [file ijms-24-10740-s001.zip › Supplementary Material S4 - Structural parameters of similarity between Ztau from human TOP2B and previosly known ZBPs .pdf]

Supplementary Material S4. Structural parameters of similarity between Zτ of human TOP2B (Q02880) and Zα/Zβ of various proteins: human ADAR1 (P55265), human ZBP1 (Q9H171), PKZ from *Danio rerio* (Q5NE12), E3 from *Vaccinia virus* (P21605), and ORF112 from *Cyprinid herpesvirus 3* (A4FTK7), together with negative controls UFM1 (P61960) and H4 (P62805) from *Homo sapiens.* p-values lower than the 0.05 threshold are in bold.

**Feature**	**Parameter**	**Units**	**Domain**								
			**Zα**	**Zβ**	**Zα1**	**Zα2**	**Zα**	**Zα**	**Zα**	**n.c.**	**n.c.**
**Protein**			ADAR1p150	ADAR1p150	ZBP1	ZBP1	PKZ	E3	ORF112	UFM1	H4
**RMSD**	structure	Å	2.4	2.7	2.6	3.2	2.5	2.6	2.9	3.4	2.8
**p-value**	structure	10^{-3}	**0.14**	**0.38**	**0.44**	**3.5**	**0.27**	**0.38**	**0.97**	196	55
**Equivalent positions**	structure	no.	64	63	60	62	60	61	61	42	51
**Gaps**	sequence	%	30	32	34	33	34	32	34	36	7
**Identity**	sequence	%	7.7	7.6	3.3	5.4	2.2	4.4	6.5	9.1	7.3
**Similarity**	sequence	%	29	24	18	19	17	19	21	23	20
